# COPD Is Associated with Elevated IFN-β Production by Bronchial Epithelial Cells Infected with RSV or hMPV

**DOI:** 10.3390/v13050911

**Published:** 2021-05-14

**Authors:** Natasha Collinson, Natale Snape, Kenneth Beagley, Emmanuelle Fantino, Kirsten Spann

**Affiliations:** 1 Centre for Immunology and Infection Control, School of Biomedical Sciences, Faculty of Health, Queensland University of Technology, Brisbane, QLD 4000, Australia; natasha.collinson@qimrberghofer.edu.au (N.C.); k2.beagley@qut.edu.au (K.B.); 2 QIMR Berghofer Medical Research Institute, Herston, QLD 4006, Australia; 3 Faculty of Medicine, Translational Research Institute, University of Queensland Diamantina Institute, Woolloongabba, QLD 4102, Australia; n.snape@uq.edu.au; 4 Children’s Health and Environment Program, Children’s Health Research Centre, The University of Queensland, Brisbane, QLD 4101, Australia; e.fantino@uq.edu.au

**Keywords:** chronic obstructive pulmonary disease, interferon, epithelial cells, metapneumovirus, respiratory syncytial virus

## Abstract

IFN treatment may be a viable option for treating COPD exacerbations based on evidence of IFN deficiency in COPD. However, in vitro studies have used primarily influenza and rhinoviruses to investigate IFN responses. This study aims to investigate the susceptibility to infection and IFN response of primary bronchial epithelial cells (BECs) from COPD donors to infection with RSV and hMPV. BECs from five COPD and five healthy donors were used to establish both submerged monolayer and well-differentiated (WD) cultures. Two isolates of both RSV and hMPV were used to infect cells. COPD was not associated with elevated susceptibility to infection and there was no evidence of an intrinsic defect in IFN production in either cell model to either virus. Conversely, COPD was associated with significantly elevated IFN-β production in response to both viruses in both cell models. Only in WD-BECs infected with RSV was elevated IFN-β associated with reduced viral shedding. The role of elevated epithelial cell IFN-β production in the pathogenesis of COPD is not clear and warrants further investigation. Viruses vary in the responses that they induce in BECs, and so conclusions regarding antiviral responses associated with disease cannot be made based on single viral infections.

## 1. Introduction

Chronic obstructive pulmonary disease (COPD) is currently the third leading cause of death worldwide [1]. An estimated 328 million people suffer from COPD, positioning this disease as a leading cause of morbidity and mortality globally [2,3]. The progression of COPD, the frequency of exacerbations requiring hospitalisation, and the commonality of co-morbidities such as heart disease affect quality of life for patients and place enormous strain on the global healthcare economy [4].

Viral infections are a major cause of hospitalisation for COPD exacerbations [5]. In healthy individuals, respiratory viral infection by seasonal influenza virus (IFV), rhinovirus (RV), respiratory syncytial virus (RSV), and human metapneumovirus (hMPV) are self-limiting, restricted to the upper airways, with few, fast-resolving symptoms. In patients with COPD, the same viral infections are more severe and more likely to spread to the lower airways, causing exacerbation of the disease [6,7,8]. IFV, RV, and RSV are the most prevalent viruses identified in clinical COPD exacerbations [6,9]. HMPV is emerging as an exacerbator of COPD, although a global neglect to test for this virus in the adult population has delayed recognition [10,11].

There are currently no specific antiviral treatments for COPD. However, recombinant IFN-β is currently in clinical trials as a treatment due to its antiviral stimulating functions [12] and has been shown to counteract the suppressive effects of inhaled glucocorticosteroid (GSC) on the antiviral response in both a mouse model and primary cell cultures [13,14]. The main driver for IFN-β as a treatment is evidence that COPD is associated with reduced IFN-β and/or IFN-λ production, making individuals more susceptible to viral infections [15,16]. However, not all studies agree [14,17], and a recent study has demonstrated delayed rather than reduced IFN responses by infected primary bronchial epithelial cells (BECs) from COPD patients [18]. Although several viruses induce exacerbation, in vitro investigations of the IFN response in COPD have primarily utilised IFV or RV. Therefore, the aim of our study was to identify if BECs from COPD patients were deficient in type I and III IFN production and more susceptible to infection by RSV and hMPV, as understudied viruses that trigger exacerbations of COPD. We also used both well-differentiated BECs at an air–liquid interface and submerged cell cultures, to identify if cell differentiation influences virus replication and the IFN response.

## 2. Materials and Methods

### 2.1. Cells and Cell Culture

Diseased Human Bronchial Epithelial (DHBE) cells from five COPD subjects, and Normal Healthy Bronchial Epithelial (NHBE) cells from five healthy subjects, were purchased from Lonza, Maryland, US. All healthy donors were non-smokers, whilst all COPD donors were smokers. The mean age (and range) was 59.3 (54–66) years for healthy and 60.3 (53–66) years for COPD donors. The COPD donors were all males, whilst 4/5 healthy donors were male.

For experimental infection of submerged monolayers, collagen-coated, 12-well culture plates were seeded with DHBE or NHBE cells at a density of 0.3 × 10^6^ cells/well. Cells were resuspended in Bronchial Epithelial Growth Medium (BEGM, Lonza) containing insulin (0.1%), gentamycin (0.1%), retinoic acid (0.1%), epinephrine (0.1%), transferrin (0.1%), triiodothyronine (0.1%), human epidermal growth factor (0.1%), and hydrocortisone (0.1%) (HC) and cultured with media, changed every 2–3 days until 50% confluent when HC was removed from the growth media. Cells were cultured for a further 24 h or until 75% confluent, prior to inoculation with virus.

For ciliated, well-differentiated (WD) cultures, cells were seeded onto collagen-coated Costar^®^ trans-well inserts (6.5mm insert, 0.4μm membrane) at 0.25 × 10^6^ cells/trans-well, in Bronchial Air–Liquid Interface (B-ALI) growth media (LonzaWhen 100% confluent; cells were differentiated at the air–liquid interface (ALI) in B-ALI differentiation media (Lonza) containing bovine pituitary extract (0.4%), insulin (0.1%), gentamycin (0.1%), retinoic acid (0.1%), epinephrine (0.1%), transferrin (0.1%), triiodothyronine (0.1%), human epidermal growth factor (0.1%) inducer, and hydrocortisone (0.1%). Cells were maintained at ALI for 3–4 weeks, until all replicate cultures were 100% ciliated and mucus was observed swirling on the apical surface. Medium was changed every 2–3 days throughout this process, mucous was removed twice weekly, and cells were washed with PBS once a week. Once fully ciliated and ready for infection, HC was removed from basal media and cells inoculated with virus 24 h later.

### 2.2. Virus Propagation and Infection

To establish consistency of antiviral response based on virus species, two strains each of hMPV and RSV were used: hMPV-CAN 97–83 (from Dr. Guy Bovin CHUQ, Quebec, QC, Canada), hMPV-AUS-001 (Queensland Paediatric Infectious Disease research group, Brisbane), RSV-A2 (ATCC), and RSV-RS4 (Prof. Paul Young, UQ). Viruses were propagated and purified through sucrose gradient as previously described [19].

Submerged cells were infected with virus at a multiplicity of infection (MOI) of 0.1 virus particles/cell, or virus-free culture media as a negative control and incubated for 2 h at 37 °C [19]. Cells were washed three times with 1× PBS and media replaced with HC-free BEGM. WD-BECs were infected with virus at a MOI of 3, or virus-free culture media as a negative control, incubated for 4 h at 37 °C, washed three times with Hanks-buffered saline solution (HBSS, Lonza), and basal media replaced with HC-free B-ALI media. MOIs used for WD-BECs were optimised in preliminary unpublished experiments, to allow maximum antiviral response with minimal cell death.

### 2.3. Virus and Interferon Quantification

Submerged cell culture supernatants were collected on 0, 1, 3, and 5 days post-infection (d.p.i.) and clarified by centrifugation prior to quantification of shed virus and IFN secretion. For WD-BECs, the apical surfaces were washed with 100μL of HBSS for 10 min every 2–3 days for 27 days, or until cultures expired.

Shed virus was quantified using a foci-forming assay for hMPV [20] and a plaque-forming assay for RSV [21] as previously described. Briefly, LLC-MK2 (hMPV) and Hep-2 cells (RSV) were infected with a 10-fold serial dilution (10^−2^ to 10^−4^) of cell supernatant/apical wash, overlayed with 0.8% methylcellulose/OptiMEM (Life Technologies), and incubated for 10 days at 32 °C (hMPV) or 7 days at 37 °C (RSV). Cells were then fixed, probed for virus-positive foci or plaques with mouse anti-hMPV-F protein antibody or goat anti-RSV polyclonal antibody, exposed to HRP-conjugated anti-mouse IgG (hMPV) or anti-goat IgG (RSV), and stained with DAB (Sigma-Aldrich). Viral titre was quantified as focus-forming units (ffu) or plaque-forming units (pfu)/mL virus suspension.

Secreted IFN-β was quantified from the same cell cultures using a 384-well AlphaLISA assay (Perkin Elmer). Secreted IFN-λ_2_ (IL28A) was quantified using a standard ELISA (Thermo-Fisher/Invitrogen) according to each manufacturer’s instructions.

### 2.4. Statistical Analysis

Grouped analysis of the data was performed using *t*-tests in GraphPad Prism (version 7) to identify statistical differences between disease states and/or viruses. *p* values less than 0.05 were considered statistically significant.

## 3. Results

### 3.1. Viral Shedding and IFN Production by Submerged BECs

**Figure 1:**
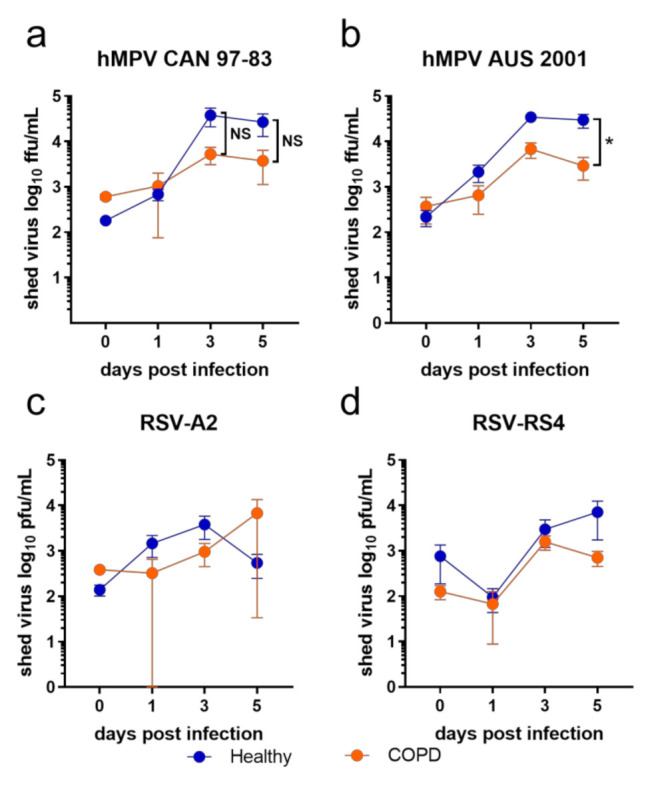
Viral shedding from submerged monolayers of BECs infected with (**a**) CAN 97-83 (**b**) AUS-001 (**c**) A2 or (**d**) RS4, MOI 0.1. Shed virus was quantified by focus/plaque assay at day 0, 1, 3, and 5 post-infection. The symbols represent the mean ± SEM. *n* = 5 for both COPD and healthy groups. Unpaired *t*-tests show statistical significance, * denotes *p* < 0.05 for COPD group compared to the healthy group.

BECs from COPD donors displayed a trend of less viral shedding compared to healthy BECs in response to all viruses tested in this study (Figure 1). This trend was clearer in response to the two hMPV isolates (Figure 1a,b; days 3 and 5) compared to the two RSV isolates—however, only hMPV AUS-001 elicited a significant difference between healthy and COPD BECs at day 5 p.i. (healthy mean = 29,723.9 ffu)/mL, COPD mean = 2920.35 ffu/mL; Figure 1b). Virus was not detected in any mock-infected wells at any time.

**Figure 2:**
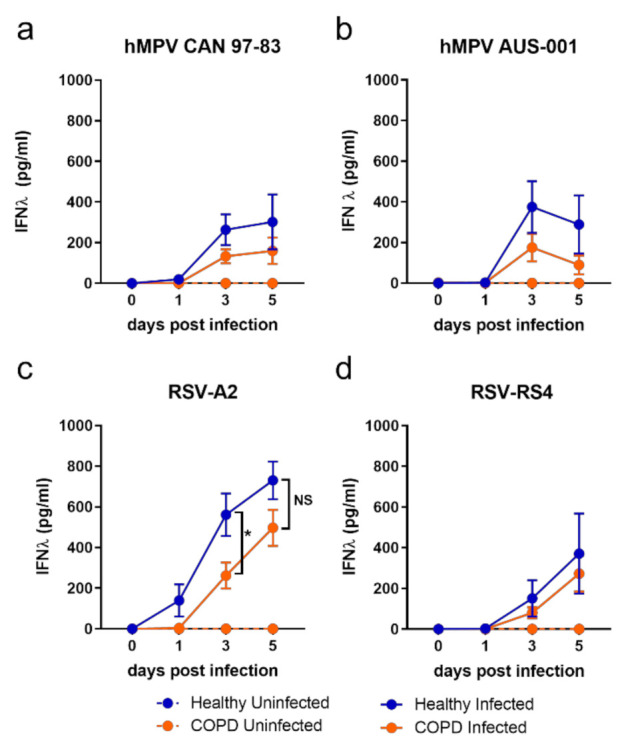
IFN-λ production by submerged monolayers of BECs infected with (**a**) CAN 97–83 (**b**) AUS-001 (**c**) A2 or (**d**) RS4, MOI 0.1. Secreted IFN-λ was quantified in cell supernatant at day 0, 1, 3, and 5 post-infection by ELISA. The symbols represent the mean ± SEM. *n* = 5 for both COPD and healthy groups. Unpaired *t*-tests show statistical significance, * denotes *p* < 0.05 for COPD group compared to the healthy group.

The kinetics of IFN-λ production in submerged monolayers were analogous to the shed virus, with a trend toward elevated IFN-λ production from healthy BECs compared to COPD in response to all viruses on days 3 and 5 p.i. (Figure 2). However, this trend reaches statistical significance only in response to RSV-A2 (Figure 2c), with healthy BECs producing significantly more IFN-λ than COPD BECs at 3 d.p.i. (healthy mean = 626.3 pg/mL, COPD mean = 262.44 pg/mL).

**Figure 3:**
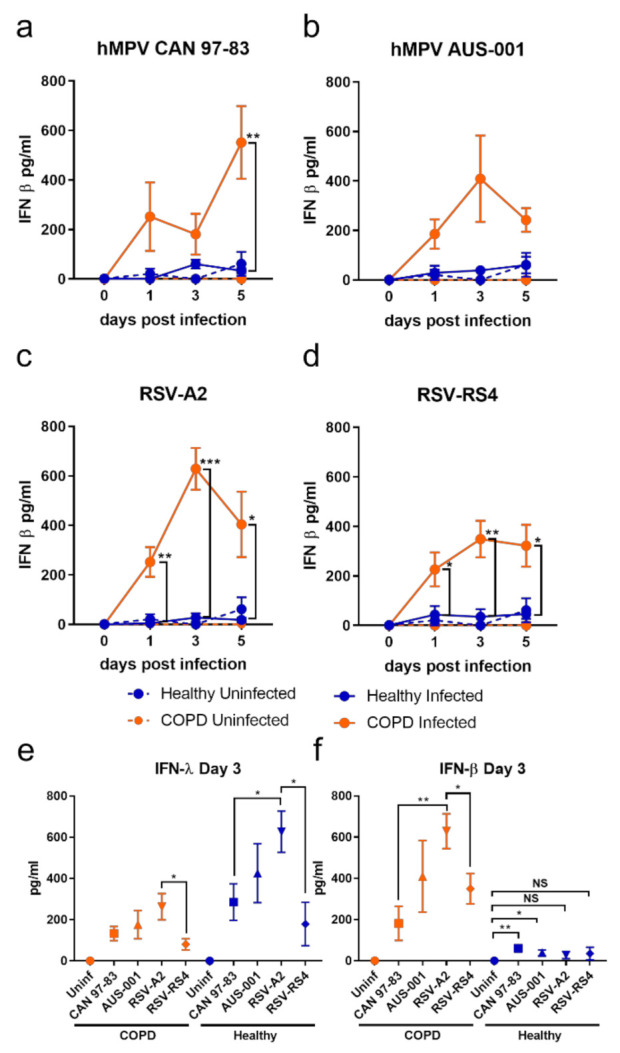
IFN-β production by submerged monolayers of BECs infected with (**a**) CAN 97-83 (**b**) AUS-001 (**c**) A2 or (**d**) RS4, MOI 0.1. Secreted IFN-β was quantified in cell supernatant at day 0, 1, 3, and 5 post-infection by AlphaLISA. The symbols represent the mean ± SEM. *n* = 5 for both COPD and healthy groups. Unpaired *t*-tests show statistical significance, * denotes *p* < 0.05 for COPD group compared to the healthy group. (**e**) IFN-λ and (**f**) IFN-β data for day 3 post-infection were extracted for comparison of production amongst viruses. Unpaired *t*-tests show statistical significance, * denotes *p* < 0.05, ** denotes *p* < 0.01, *** *p* < 0.001.

Contrary to IFN-λ, IFN-β production was significantly elevated in COPD BECs in response to all viruses (Figure 3). This trend was clearer in response to RSV (Figure 3c,d) compared to hMPV (Figure 3a,b), with a statistical difference in IFN-β for COPD compared to health BECs reached on days 1, 3, and 5 p.i. for RSV, compared to only on 5 d.p.i. in response to CAN-97-83.

Examining IFN production data at day 3 p.i. only, a differential response associated with disease was clear: healthy BECs favoured IFN-λ production and COPD BECs favoured IFN-β production (Figure 3e,f). It was also evident at 3 d.p.i. that some viral strains were stronger inducers of IFN than others. The lab-adapted RSV-A2 strain induced more IFN than the RS4 strain, and a pattern of strongest-to-weakest IFN-λ induction—A2 > AUS-001 > CAN 97-83 > RS4—by both COPD and healthy BECs was apparent. A similar pattern at 3 d.p.i. was observed for IFN-β production in COPD BECs subjects, although CAN 97-83 induced the least IFN-β in COPD BECs.

### 3.2. Viral Shedding and IFN Production by WD-BECs

**Figure 4:**
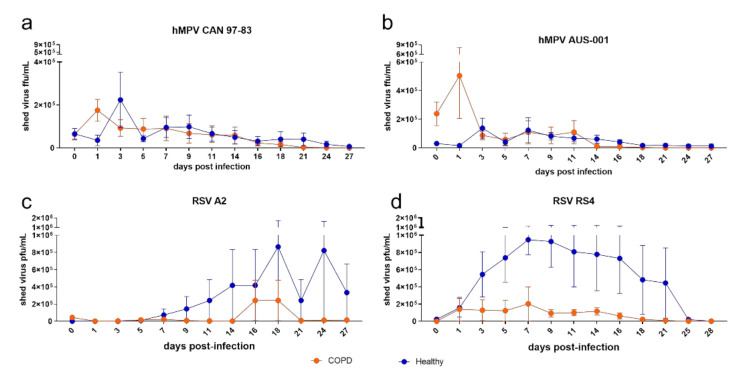
Virus shed by well-differentiated BEC cultures infected with (**a**) CAN 97-83 (**b**) AUS-001 (**c**) A2 or (**d**) RS4, MOI 3. The apical surface was washed every 2–3 days for 27 days and shed virus quantified by focus/plaque assay. The symbols represent the mean ± SEM. *n* = 5 for both COPD and healthy groups. Unpaired *t*-tests were performed, no statistically significant differences were observed.

To ensure that the data generated from submerged cultures were robust, we established WD-BECs using the same subjects and maintained them over a longer time period (27 days p.i.) to allow for slower virus replication and cell-to-cell infection. As observed for submerged monolayers, COPD was not associated with significantly elevated shed virus (Figure 4). For hMPV, virus shed from healthy and COPD BECs was comparable, apart from an early spike in AUS-001 shed from COPD BECs at 1 d.p.i. (Figure 4b). The kinetics of RSV (Figure 4c,d) shedding were noticeably different to hMPV, with a strong trend for healthy BECs to shed more virus than COPD BECs over the 27 days of quantification. For RS4, this was observed from 3 to 24 d.p.i., with peak shedding from healthy BECs on day 7 (mean 948,375 pfu/mL). In COPD BECs, RS4 levels remained low, with no shed virus detected beyond 18 d.p.i. A2 shedding from healthy BECs was delayed relative to RS4, with notable peaks on days 18 (mean 866,711 pfu/mL) and 24 p.i. (mean 822,617 pfu/mL). Shed A2 was undetectable in COPD BEC cultures until 16 d.p.i., 9 days later than for healthy BECs, with no detectable virus shed after 21 d.p.i.

**Figure 5:**
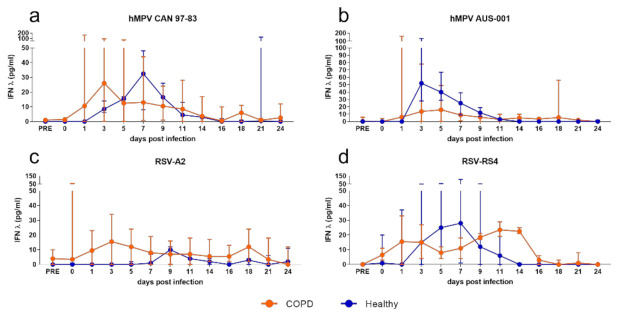
IFN-λ production by well-differentiated BEC cultures infected with (**a**) CAN 97-83 (**b**) AUS-001 (**c**) A2 or (**d**) RS4, MOI 3. Basal medium was sampled 3 times per week and secreted IFN-λ quantified by ELISA. The symbols represent the mean ± SEM. *n* = 5 for both COPD and healthy groups. Unpaired *t*-tests were performed, no statistically significant differences were observed.

**Figure 6:**
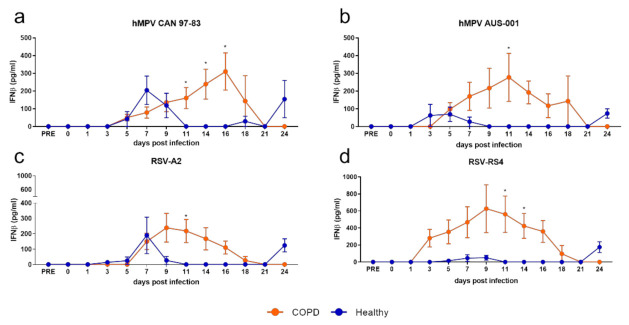
IFN-β production by well-differentiated BEC cultures infected with (**a**) CAN 97-83 (**b**) AUS-001 (**c**) A2 or (**d**) RS4, MOI 3. Basal medium was sampled 3 times per week and secreted IFN-β quantified by AlphaLISA. The symbols represent the mean ± SEM. *n* = 5 for both COPD and healthy groups. Unpaired *t*-tests show statistical significance, * denotes *p* < 0.05.

In response to both hMPV (Figure 5a,b) and RSV (Figure 5c,d), healthy and COPD WD-BECs produced very little IFN-λ (mean 50 pg/mL or less), with no association between COPD and modulated IFN-λ production. Conversely, and similarly to what was observed in submerged cultures, WD-BECs from COPD subjects produced significantly more IFN-β in response to hMPV and RSV than healthy WD-BECs (Figure 6). In response to both hMPV viruses, COPD WD-BECs produced detectable IFN-β from 5 to 18 d.p.i., reaching similar peak means at 15 d.p.i. for CAN 97-83 (310 pg/mL; Figure 6a) and 11 d.p.i. for AUS-(277 pg/mL; Figure 6b). Production of IFN-β in response to A2 was elevated and sustained for longer by COPD compared to healthy WD-BECs. Of the four viruses, RS4 elicited the earliest detected (3 d.p.i.) and highest IFN-β production from COPD WD-BECs, peaking at 9 d.p.i. (mean 626 pg/mL; Figure 6d) and reaching statistical significance over healthy WD-BEC production at 11–13 d.p.i. Overall, the WD-BEC data correlated to the submerged culture model in that COPD was associated with elevated IFN-β production compared to healthy cells.

## 4. Discussion

COPD patients are highly susceptible to respiratory virus infections [22,23] that exacerbate disease [24]. The exact role of the antiviral response of the airway epithelium in COPD is not clear, with some research identifying innate immune defects associated with COPD [22,25]; conversely, others have identified elevated antiviral factors, such as IFN-λ, associated with viral infections in COPD [26,27]. There is almost no research concerning the response of COPD patients to viruses other than RV and influenza, although many viruses cause acute exacerbations of COPD. Disease-based generalisations regarding antiviral response founded on only one virus are not always reliable. Here, we used four different isolates of two pneumoviruses, in two cellular models, to investigate potential differences in the antiviral response based on the infecting virus in addition to disease.

Regardless of the virus strain and cellular model used in this study, BECs from COPD subjects were not more susceptible to infection than healthy BECs. This is consistent with other studies in which either submerged [17] or WD [18] BECs from asthmatic or COPD patients were exposed to RV-1B or RV-A1, respectively, and viral replication quantified up to 7 days p.i. In contrast, an earlier study, also using WD-BECs infected with RV-39, demonstrated elevated virus replication in COPD cells compared to healthy cells 24 and 48 h.p.i. [23]. Very few studies have used hMPV in experimental infection. However, a study by Kan-O et al. (2017) found elevated hMPV and N gene expression in submerged BECs from COPD subjects, compared to healthy subjects, at 48 h and 72 h.p.i. [14]. A study by Hsu et al. (2016) demonstrated elevated influenza H5N1 viral load in COPD BECs compared to healthy cells 24 h.p.i. [28]. The muted susceptibility of COPD BECs to RSV infection observed in this study may align with epidemiological data that suggest that RSV is only identified in up to 15% COPD exacerbations, significantly less than RV or influenza virus [29].

This may be due to differences in virus receptor expression on the surfaces of cells from COPD patients. For RSV, nucleolin [30] and insulin-like growth factor-1 receptor (IGF1R) [31] are key binding receptors. However, how their expression may be modified in COPD is unknown. Susceptibility of COPD BECs to infection differs relative to the infecting virus, and so generalisations concerning the antiviral response cannot be made. Future studies dissecting the mechanisms of infection and viral entry specific to disease state are warranted, so that a deeper understanding of cellular response can be acquired.

In addition, it needs to be acknowledged that differences between our observations and other published studies may be due, in part, to differences in the source of cells. Donor variability always needs to be considered when comparing studies, as do commercial sources of cells (although still primary cells) compared with primary cells sampled directly from donors.

The IFN response of BECs was investigated to identify any potential defects in the antiviral response to RSV or hMPV associated with COPD. Submerged COPD BECs demonstrated a trend towards reduced IFN-λ protein secretion in comparison to healthy BECs. Virus shedding was, however, also reduced in these same COPD cells, which suggests that IFN-λ production is associated with virus production and that there is no intrinsic defect in IFN-λ production associated with COPD. We have previously demonstrated that AECS in adults with asthma [20] and children with wheeze [19] do not have any intrinsic defects in the IFN response to RSV or hMPV.

Interestingly, our study demonstrated elevated IFN-β protein production in association with COPD in response to all four viruses and in both culture models. This has been reported previously in response to hMPV infection [14] and RV-1B infection [17]. In contrast, COPD BECs infected with different strains of influenza [17,28] or RV-39 [23] exhibited reduced IFN-β production [22]. For HMPV, these data suggest that similar (for WD-BECS) or even lower (for submerged cultures) levels of virus production and spread within COPD compared to healthy BECs stimulate a more exuberant IFN-β response from COPD BECs than from healthy BECs. This may suggest a parallel hyperinflammatory response to HMPV infection associated with COPD, which warrants further investigation.

For RSV, the relationship between virus shedding and IFN-β production is similar to that of HMPV in submerged cultures, with comparable levels of virus shedding resulting in significantly more IFN-β production associated with COPD. For WD-BECs infected with RSV, however, the relationship between virus and IFN-β production is less clear and may be the result of more complex differences in the response of healthy and COPD BECs. For example, the highly effective ability of RSV to suppress IFN-β production and signalling [21] may explain elevated viral replication and reduced IFN-β production in healthy BECs. However, this does not seem to occur in COPD BECs. Suppressed virus production may be the result of reduced cell surface receptors, as discussed earlier, or modulated virus release mechanisms, which suggests that COPD cells are morphologically or physiologically different to healthy cells, such that the kinetics of RSV infection are modulated. Regardless of viral kinetics, though, any ability of RSV to suppress IFN-β production appears to be overwhelmed in BECs from COPD donors. Similarly to the response to hMPV, this may suggest a parallel hyperinflammatory response, which requires further investigation and may provide further insight into the pathogenesis of COPD exacerbation by viral infections [32]. The clinical implications of elevated IFN-β production in BECs from COPD patients warrant further investigation, as it may underlie elevated susceptibility to secondary bacterial infections. IAV-induced IFN-β promotes *Streptococcus pneumonia* colonisation in mice [33], and RSV-induced IFN-β enhances *Pseudomonas aeruginosa* biofilm formation in the airway epithelium through dysregulated iron homeostasis [34]. IFN signalling also shapes the inflammatory response to respiratory infections and promotes inflammation-induced disease, particularly in response to RSV infection [32,35]. Therefore, although COPD is not associated with elevated RSV or hMPV infection, disease exacerbation may be driven by excessive IFN-β production by BECs. The exact mechanism by which this occurs warrants further investigation, as COPD-specific differences in cell surface receptor expression, cell death responses, cell morphology and function within the epithelial layer, and barrier function may all influence the IFN-driven response to different viruses in different ways. Changes in secretory cell development, ciliation, and basal cell morphology in the airways of patients with COPD have been reported [36]. From this study and others, it is clear that a one-size-fits-all view of antiviral responses of the airway epithelium in COPD is not viable.

## Data Availability

The data set analysed is available from the authors upon reasonable request.
